# Supplementary material for: Regulated changes in material properties underlie centrosome disassembly during mitotic exit
Source: J Cell Biol. 2020 Feb 12;219(4):e201912036. doi: 10.1083/jcb.201912036 (PMC7147112; doi:10.1083/jcb.201912036)
Supplement: Table S1 — lists C. elegans strains used in this study. [file JCB_201912036_TableS1.docx]

**TABLE S1. *C. elegans* strains used in this study**

| Strain name | genotype | Creation method | Origin |
| --- | --- | --- | --- |
| DAM858 | vie11[pAD676; gfp::tac-1]II | CRISPR | Alexander Dammermann |
| EU584 | spd-2(or188ts) I | mutagenesis | Bruce Bowerman |
| JWW1 | utsw2[mMaple::spd-5] I | CRISPR | This study |
| JWW13 | spd-2(or188ts) I; unc-119(ed9) III; ltSi202[pVV103/ pOD1021; Pspd-2::GFP::SPD-5 RNAiresistant;cb-unc-119(+)]II | Cross of EU584 and OD847 | This study |
| JWW35 | ltSi202[pVV103/ pOD1021; Pspd-2::GFP::SPD-5 RNAiresistant;cb-unc-119(+)]II ; unc-119(ed3)III; ddIs44[WRM0614cB02 GLCherry::tbg-1;Cbr-unc-119(+)] | Cross of OD847 and TH169 | This study |
| JWW64 | utsw2[mMaple::spd-5] I; ltIs37 [(pAA64) pie-1p::mCherry::his-58 + unc-119(+)] IV. | Cross of JWW1 and OD95 | This study |
| JWW65 | lt17[plk-1::gfp+loxP]III ; ltIs37 [(pAA64) pie-1p::mCherry::his-58 + unc-119(+)] IV; unc-119(ed3) III | Cross of OD2425 and OD95 | This study |
| JWW66 | ltSi203[pVV60; Pspd-2::GFP::SPD-2 reencoded; cb-unc-119(+)]II; ltIs37 [(pAA64) pie-1p::mCherry::his-58 + unc-119(+)] IV; unc-119(ed3) III | Cross of OD824 and OD95 | This study |
| JWW67 | unc-119(ed9) III; utsw1[pJWB56; Pspd-2::GFP::SPD-5(530E, 627E, 653E, 658E) re-encoded; cb-unc-119(+)]II | MosSCI, into EG6699 | This study |
| JWW69 | unc-119(ed9) III; ltSi202[pVV103/ pOD1021; Pspd-2::GFP::SPD-5 RNAiresistant;cb-unc-119(+)]II; ltIs37 [(pAA64) pie-1p::mCherry::his-58 + unc-119(+)] IV. | Cross of OD847 and OD95 | This study |
| JWW70 | unc-119(ed9) III; utsw1[pJWB56; Pspd-2::GFP::SPD-5(530E, 627E, 653E, 658E) re-encoded; cb-unc-119(+)]II; ltIs37 [(pAA64) pie-1p::mCherry::his-58 + unc-119(+)] IV. | Cross of JWW1 and OD95 | This study |
| JWW71 | lt17[plk-1::gfp+loxP]III; unc-119(ed3)III; ddIs44[WRM0614cB02 GLCherry::tbg-1;Cbr-unc-119(+)] | Cross of OD2425 and TH169 | This study |
| JWW72 | vie11[pAD676; gfp::tac-1]II ; unc-119(ed3)III; ddIs44[WRM0614cB02 GLCherry::tbg-1;Cbr-unc-119(+)] | Cross of DAM858 and TH169 | This study |
| JWW89 | spd-2(or188ts) I; ltSi202[pVV103/ pOD1021; Pspd-2::GFP::SPD-5 RNAiresistant;cb-unc-119(+)]II; ltIs37 [(pAA64) pie-1p::mCherry::his-58 + unc-119(+)] IV. | Cross of JWW13 and OD95 | This study |
| OD2425 | lt17[plk-1::gfp+loxP]III | CRISPR | Karen Oegema |
| OD823 | ltSi203[pVV60; Pspd-2::GFP::SPD-2 reencoded; cb-unc-119(+)]II; unc-119(ed3) III | MosSCI, into EG6699 | Karen Oegema |
| OD847 | unc-119(ed9) III; ltSi202[pVV103/ pOD1021; Pspd-2::GFP::SPD-5 RNAiresistant;cb-unc-119(+)]II | MosSCI, into EG6699 | (Woodruff et al., 2015) |
| OD95 | unc-119(ed3) III; ltIs37 [(pAA64) pie-1p::mCherry::his-58 + unc-119(+)] IV; ltIs38 [pie-1p::GFP::PH(PLC1delta1) + unc-119(+)] | Microparticle bombardment | CGC |
| TH169 | unc-119(ed3)III; ddIs44[WRM0614cB02 GLCherry::tbg-1;Cbr-unc-119(+)] | Microparticle bombardment | Anthony Hyman |
| TH447 | unc-119(ed9) III; ddIs243[pie-1p::LAP::LET-92; unc-119(+)]; ddIs247[pie-1p::SPD-5(synthetic introns, CAI 0.65)::mCherry; unc-119(+)] | Microparticle bombardment | Anthony Hyman |
| TH530 | rsa-1::LAP; unc-119(ed3)III; ddIs44[WRM0614cB02 GLCherry::tbg-1;Cbr-unc-119(+)] | Microparticle bombardment | Anthony Hyman |
| TH531 | rsa-2::LAP; unc-119(ed3)III; ddIs44[WRM0614cB02 GLCherry::tbg-1;Cbr-unc-119(+)] | Microparticle bombardment | Anthony Hyman |
| TH539 | spd-2::GFP; unc-119(ed3)III; ddIs44[WRM0614cB02 GLCherry::tbg-1;Cbr-unc-119(+)] | Microparticle bombardment | Anthony Hyman |
| TH571 | unc-119(ed3)III; ddIs12[pie-1p::tpxl-1::GFP;unc-119(+)]; ddIs44[WRM0614cB02 GLCherry::tbg-1;Cbr-unc-119(+)] | Microparticle bombardment | Anthony Hyman |
| TH630 | ddIs44[WRM0614cB02 GLCherry::tbg-1;Cbr-unc-119(+)]; ddIs62[pie-1p::AIR-1(synthetic introns, CAI 1.0)::GFP; unc-119(+)]; unc-119(ed3)III | Microparticle bombardment | Anthony Hyman |
| EG6699 | ttTi5605 II; unc-119(ed3) III; oxEx1578. |  | CGC |
