# Supplementary material for: Regulated changes in material properties underlie centrosome disassembly during mitotic exit
Source: J Cell Biol. 2020 Feb 12;219(4):e201912036. doi: 10.1083/jcb.201912036 (PMC7147112; doi:10.1083/jcb.201912036)
Supplement: Table S2 — lists protein expression plasmids used in this study. [file JCB_201912036_TableS2.docx]

**TABLE S2. Protein expression plasmids used in this study**

| Plasmid name | Gene | N-term tag | C-term tag | Origin |
| --- | --- | --- | --- | --- |
| JWV11 | *plk-1(T194D)*  constitutively active |  | PreScission-6xHis | (Woodruff et al., 2015) |
| JWV12 | *plk-1(K67M)*  kinase dead |  | PreScission-6xHis | (Woodruff et al., 2015) |
| JWV2 | *spd-5(wt)* | MBP-PreScission | PreScission-6xHis | (Woodruff et al., 2015) |
| JWV3 | *spd-5(wt)* | MBP-PreScission | tagRFP-PreScission-6xHis | (Woodruff et al., 2015) |
| JWV6 | *spd-2(wt)* | MBP-TEV | TEV-6xHis | (Woodruff et al., 2015) |
