# Supplementary material for: Regulated changes in material properties underlie centrosome disassembly during mitotic exit
Source: J Cell Biol. 2020 Feb 12;219(4):e201912036. doi: 10.1083/jcb.201912036 (PMC7147112; doi:10.1083/jcb.201912036)
Supplement: Table S3 — shows one-way ANOVA and post hoc tests of anterior PCM disassembly profiles from Fig. 3 F. [file JCB_201912036_TableS3.docx]

**TABLE S3. One-way ANOVA and post-hoc tests of anterior PCM disassembly profiles from Figure 3F**

| Holm-Sidak's multiple comparisons test | Mean Diff. | Significant? | Summary | Adjusted P Value |
| --- | --- | --- | --- | --- |
| AIR-1 vs. TPXL-1 | -25.55 | No | ns | 0.2725 |
| AIR-1 vs. RSA-1 | -8.310 | No | ns | 0.9615 |
| AIR-1 vs. RSA-2 | -14.38 | No | ns | 0.8174 |
| AIR-1 vs. SPD-2 | 38.26 | Yes | ** | 0.0079 |
| AIR-1 vs. SPD-5 | -24.73 | No | ns | 0.2183 |
| AIR-1 vs. TBG-1 | 8.728 | No | ns | 0.9598 |
| AIR-1 vs. TAC-1 | 0.4412 | No | ns | 0.9963 |
| AIR-1 vs. PLK-1 | 92.29 | Yes | **** | <0.0001 |
| TPXL-1 vs. RSA-1 | 17.24 | No | ns | 0.8060 |
| TPXL-1 vs. RSA-2 | 11.16 | No | ns | 0.9598 |
| TPXL-1 vs. SPD-2 | 63.81 | Yes | **** | <0.0001 |
| TPXL-1 vs. SPD-5 | 0.8171 | No | ns | 0.9963 |
| TPXL-1 vs. TBG-1 | 34.28 | Yes | * | 0.0356 |
| TPXL-1 vs. TAC-1 | 25.99 | No | ns | 0.4307 |
| TPXL-1 vs. PLK-1 | 117.8 | Yes | **** | <0.0001 |
| RSA-1 vs. RSA-2 | -6.074 | No | ns | 0.9615 |
| RSA-1 vs. SPD-2 | 46.57 | Yes | ** | 0.0012 |
| RSA-1 vs. SPD-5 | -16.42 | No | ns | 0.8060 |
| RSA-1 vs. TBG-1 | 17.04 | No | ns | 0.7693 |
| RSA-1 vs. TAC-1 | 8.751 | No | ns | 0.9615 |
| RSA-1 vs. PLK-1 | 100.6 | Yes | **** | <0.0001 |
| RSA-2 vs. SPD-2 | 52.64 | Yes | **** | <0.0001 |
| RSA-2 vs. SPD-5 | -10.35 | No | ns | 0.9598 |
| RSA-2 vs. TBG-1 | 23.11 | No | ns | 0.2887 |
| RSA-2 vs. TAC-1 | 14.83 | No | ns | 0.8971 |
| RSA-2 vs. PLK-1 | 106.7 | Yes | **** | <0.0001 |
| SPD-2 vs. SPD-5 | -62.99 | Yes | **** | <0.0001 |
| SPD-2 vs. TBG-1 | -29.53 | No | ns | 0.0940 |
| SPD-2 vs. TAC-1 | -37.82 | Yes | * | 0.0454 |
| SPD-2 vs. PLK-1 | 54.03 | Yes | *** | 0.0002 |
| SPD-5 vs. TBG-1 | 33.46 | Yes | * | 0.0207 |
| SPD-5 vs. TAC-1 | 25.17 | No | ns | 0.4085 |
| SPD-5 vs. PLK-1 | 117.0 | Yes | **** | <0.0001 |
| TBG-1 vs. TAC-1 | -8.286 | No | ns | 0.9615 |
| TBG-1 vs. PLK-1 | 83.56 | Yes | **** | <0.0001 |
| TAC-1 vs. PLK-1 | 91.85 | Yes | **** | <0.0001 |
