# Supplementary material for: Regulated changes in material properties underlie centrosome disassembly during mitotic exit
Source: J Cell Biol. 2020 Feb 12;219(4):e201912036. doi: 10.1083/jcb.201912036 (PMC7147112; doi:10.1083/jcb.201912036)
Supplement: Table S4 — shows one-way ANOVA and post hoc tests of posterior PCM disassembly profiles from Fig. 3 G. [file JCB_201912036_TableS4.docx]

**TABLE S4. One-way ANOVA and post-hoc tests of posterior PCM disassembly profiles from Figure 3G**

| Holm-Sidak's multiple comparisons test | Mean Diff. | Significant? | Summary | Adjusted P Value |
| --- | --- | --- | --- | --- |
| AIR-1 vs. TPXL-1 | -9.235 | No | ns | 0.9002 |
| AIR-1 vs. RSA-1 | -36.70 | Yes | *** | 0.0003 |
| AIR-1 vs. RSA-2 | -46.04 | Yes | **** | <0.0001 |
| AIR-1 vs. SPD-2 | 5.014 | No | ns | 0.9652 |
| AIR-1 vs. SPD-5 | -42.30 | Yes | **** | <0.0001 |
| AIR-1 vs. TBG-1 | -13.68 | No | ns | 0.5679 |
| AIR-1 vs. TAC-1 | -34.04 | Yes | ** | 0.0041 |
| AIR-1 vs. PLK-1 | 40.38 | Yes | **** | <0.0001 |
| TPXL-1 vs. RSA-1 | -27.46 | Yes | * | 0.0334 |
| TPXL-1 vs. RSA-2 | -36.81 | Yes | *** | 0.0006 |
| TPXL-1 vs. SPD-2 | 14.25 | No | ns | 0.6655 |
| TPXL-1 vs. SPD-5 | -33.07 | Yes | ** | 0.0028 |
| TPXL-1 vs. TBG-1 | -4.446 | No | ns | 0.9652 |
| TPXL-1 vs. TAC-1 | -24.80 | No | ns | 0.1399 |
| TPXL-1 vs. PLK-1 | 49.61 | Yes | **** | <0.0001 |
| RSA-1 vs. RSA-2 | -9.346 | No | ns | 0.9002 |
| RSA-1 vs. SPD-2 | 41.71 | Yes | **** | <0.0001 |
| RSA-1 vs. SPD-5 | -5.608 | No | ns | 0.9652 |
| RSA-1 vs. TBG-1 | 23.02 | No | ns | 0.0790 |
| RSA-1 vs. TAC-1 | 2.661 | No | ns | 0.9652 |
| RSA-1 vs. PLK-1 | 77.07 | Yes | **** | <0.0001 |
| RSA-2 vs. SPD-2 | 51.06 | Yes | **** | <0.0001 |
| RSA-2 vs. SPD-5 | 3.739 | No | ns | 0.9652 |
| RSA-2 vs. TBG-1 | 32.36 | Yes | ** | 0.0014 |
| RSA-2 vs. TAC-1 | 12.01 | No | ns | 0.8399 |
| RSA-2 vs. PLK-1 | 86.42 | Yes | **** | <0.0001 |
| SPD-2 vs. SPD-5 | -47.32 | Yes | **** | <0.0001 |
| SPD-2 vs. TBG-1 | -18.70 | No | ns | 0.2609 |
| SPD-2 vs. TAC-1 | -39.05 | Yes | ** | 0.0014 |
| SPD-2 vs. PLK-1 | 35.36 | Yes | ** | 0.0020 |
| SPD-5 vs. TBG-1 | 28.62 | Yes | ** | 0.0061 |
| SPD-5 vs. TAC-1 | 8.269 | No | ns | 0.9307 |
| SPD-5 vs. PLK-1 | 82.68 | Yes | **** | <0.0001 |
| TBG-1 vs. TAC-1 | -20.35 | No | ns | 0.2689 |
| TBG-1 vs. PLK-1 | 54.06 | Yes | **** | <0.0001 |
| TAC-1 vs. PLK-1 | 74.41 | Yes | **** | <0.0001 |
