# Supplementary material for: Regulated changes in material properties underlie centrosome disassembly during mitotic exit
Source: J Cell Biol. 2020 Feb 12;219(4):e201912036. doi: 10.1083/jcb.201912036 (PMC7147112; doi:10.1083/jcb.201912036)
Supplement: Table S5 — lists oligonucleotides for creation of mMaple::SPD-5. [file JCB_201912036_TableS5.docx]

**TABLE S5. Oligos for creation of mMaple::SPD-5**

| **Name** | **Primer sequence** | **Direction** | **Step** |
| --- | --- | --- | --- |
| oJBW120 | \| cgaacccgtttcttgtttcagaaaacttcgcgttaaATGATCTCCAAGGGAGAGG \| \| --- \| | forward | inside |
| oJBW121 | \| GAGATTGGAGTCCTCGTTGAGCACAGAATTATCCTCCAT  ggatcctcctcctccCTTG \| \| --- \| | reverse | inside |
| oJBW122 | \| cgaacccgtttcttgtttca \| \| --- \| | forward | outside |
| oJBW123 | \| GAGATTGGAGTCCTCGTTGA \| \| --- \| | reverse | outside |
